# Photosynthetic response of Persian Gulf acroporid corals to summer versus winter temperature deviations

**DOI:** 10.7717/peerj.1062

**Published:** 2015-06-30

**Authors:** Jahangir Vajed Samiei, Abolfazl Saleh, Ali Mehdinia, Arash Shirvani, Mohsen Kayal

**Affiliations:** 1 Iranian National Institute for Oceanography and Atmospheric Science, Tehran, Iran; 2 Bren School of Environmental Science and Management, University of California, Santa Barbara, CA, USA

**Keywords:** Coral reefs, Global warming, Thermal tolerance, Seasonal performance

## Abstract

With on-going climate change, coral susceptibility to thermal stress constitutes a central concern in reefconservation. In the Persian Gulf, coral reefs are confronted with a high seasonal variability in water temperature, and both hot and cold extremes have been associated with episodes of coral bleaching and mortality. Using physiological performance as a measure of coral health, we investigated the thermal susceptibility of the common acroporid, *Acropora downingi*, near Hengam Island where the temperature oscillates seasonally in the range 20.2–34.2 °C. In a series of two short-term experiments comparing coral response in summer versus winter conditions, we exposed corals during each season (1) to the corresponding seasonal average and extreme temperature levels in a static thermal environment, and (2) to a progressive temperature deviation from the annual mean toward the corresponding extreme seasonal value and beyond in a dynamic thermal environment. We monitored four indictors of coral physiological performance: net photosynthesis (Pn), dark respiration (R), autotrophic capability (Pn/R), and survival. Corals exposed to warming during summer showed a decrease in net photosynthesis and ultimately died, while corals exposed to cooling during winter were not affected in their photosynthetic performance and survival. Coral autotrophic capability Pn/R was lower at the warmer thermal level within eachseason, and during summer compared to winter. Corals exposed to the maximum temperature of summer displayed Pn/R < 1, inferring that photosynthetic performance could not support basal metabolic needs under this environment. Our results suggest that the autotrophic performance of the Persian Gulf *A. downingi* is sensitive to the extreme temperatures endured in summer, and therefore its populations may be impacted by future increases in water temperature.

## Introduction

Increasing evidence of climate change toward a warmer environment with more frequent thermal anomalies points to the importance of assessing thermal susceptibility of corals in reef conservation (Berkelmans, 2002; Hoegh-Guldberg et al., 2014). Indeed, severe deviations in water temperature negatively affect physiological performance of scleractinian corals during both cold and warm seasons (Lesser, 1996; Saxby, Dennison & Hoegh-Guldberg, 2003; Roth, Goericke & Deheyn, 2012; Hoogenboom et al., 2012; Roth & Deheyn, 2013), causing widespread coral bleaching and mortality, and threatening tropical reefs (Hoegh-Guldberg et al., 2005; Lirman et al., 2011; Eakin, Kleypas & Hoegh-Guldberg, 2008). Thermal tolerance is, however, variable from one coral species to another and among regions (Jokiel & Coles, 1990; Marshall & Baird, 2000); therefore, assessment of thermal susceptibility of major reef-building corals from multiple regions to evaluate vulnerability of reef ecosystems to climate change is a research priority.

Coral reefs from the Persian Gulf subsist in a singular highly oscillating environment that in the summer constitutes the world’s warmest sea, and in the winter one of the coldest seas hosting coral reefs (Kleypas, McManus & Meñez, 1999; Sheppard et al., 2010). Therefore, estimating the thermal sensitivity of Persian Gulf corals is important for more effective reef conservation in the region. It can also benefit our general understanding of the ability of corals to adapt or acclimate to temperature variations by providing quantitative information on the limits of coral performance in populations exposed to recurring thermal stress (Feary et al., 2013). Records of thermal thresholds at occurrences of bleaching events on some southern Persian Gulf reefs indicated that *Acropora* bleaching is associated with 3 weeks of exposure to >35 °C average daily temperatures in summer (Riegl et al., 2012), and with more than 4 weeks of exposure to <13 °C average daily temperatures in winter (Coles & Fadlallah, 1991). The thermal threshold of bleaching, however, does not give much information about the effects of the non-bleaching range of temperature on physiological performance of corals, and cannot be generalized to reef areas where records of water temperature and bleaching events are scarce.

Previous studies have indicated that metabolic performance is an accurate indicator of coral thermal tolerance (reviewed by Castillo & Helmuth, 2005). Photosynthesis by symbiotic zooxanthellae is the primary process providing energy for tropical and subtropical reef-building corals (Chalker & Taylor, 1975). Both supra- and sub-optimal water temperatures disturb the photosynthetic apparatus of zooxanthellae and negatively impact coral net photosynthesis (Pn) (Lesser, 1996; Jones et al., 1998; Tchernov et al., 2004; Saxby, Dennison & Hoegh-Guldberg, 2003; Al-Horani, 2005). Besides, increases in temperature elevate respiration (R) by the holobiont cause a decrease in the autotrophic capacity (Pn/R ratio) of the coral (Castillo & Helmuth, 2005). At extreme conditions, low rates of Pn/R may restrict the capacity of the coral to build and maintain enough energy reserves (e.g., lipids) for sustaining essential homeostatic functions, including sexual reproduction and biophysical stress resistance (Wooldridge, 2014).

In this study, we experimentally evaluated the effects of seasonal temperature variations on metabolic performance and survival of a common acroporid coral from the Persian Gulf, *Acropora downingi*, collected near Hengam Island where no fatal coral bleaching has ever been documented (Vajed Samiei et al., 2014). We implemented two short-term experiments to quantify and compare coral performance under summer versus winter thermal conditions. In each season, we exposed freshly collected branchlets of *Acropora downingi* from the natural environment (1) to the average and extreme temperature levels of the season in a static thermal environment, and (2) to a progressive temperature deviation from the annual mean toward the corresponding seasonal thermal extreme. We evaluated coral physiological response under the different thermal environments by measuring and comparing net photosynthesis (Pn), dark respiration (R), net photosynthesis to respiration ratio (Pn/R), and survival. Our results suggest that Hengam Island’s *A. downingi* populations are living close to their superior thermal threshold, and thus would be affected by a warmer climate.

## Materials and Methods

### Sampling site and procedure

Our sampling site is located near Hengam Island in the north-eastern section of the Persian Gulf. Hengam reef is washed by water currents coming through the Strait of Hormuz from the Oman Sea and, hence, experiences milder seasonal water temperatures compared to reefs situated more inward in the Persian Gulf. The minimum, mean and maximum annual water temperatures at our sampling site are respectively 20.2, 27.5 and 34.2 °C; and the mean daily water temperatures during the warm and cold seasons are respectively 32.4 and 21.6 °C (Vajed Samiei et al., 2014). During the warm season, tidal flows frequently impose short-term temperature variations as large as 5.5 °C in the course of 1 to 3 h on the Hengam coral community (Vajed Samiei et al., 2014).

We used *Acropora downingi* as a study model because it dominates coral patches around Hengam Island and is one of the major reef-building corals in the Persian Gulf (Rahmani et al., 2013). In each season, experiments were performed on a daily basis on two 10–15 cm branches of *A. downingi* collected from a depth of about 4 m and transported in seawater and low light conditions to the nearby laboratory within 15 min. Samples were kept immersed at the annual average thermal level of 27.5 °C in aerated seawater for 3 h prior to experiments.

### Experimental set up and measurements

**Figure 1:**
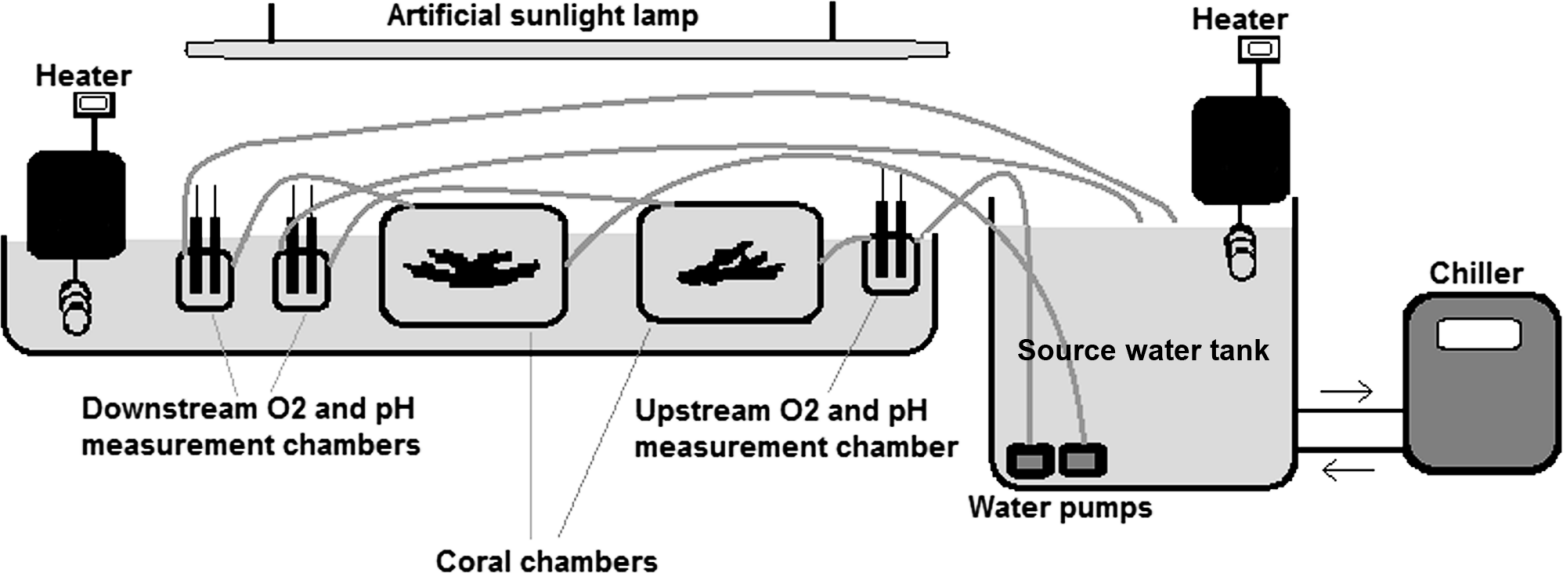
The experimental set up. Seawater collected from the study site was pumped from the source-water tank through the system at a constant rate of about 0.5 l. min^−1^. Oxygen concentration (mg. l^−1^) and pH were recorded upstream and downstream the coral chambers every 5 min. 6,500 lux light (i.e. about 120 µ mol. m^−2^ .s^−1^) was provided by an artificial sunlight lamp (Dymax Rex-2). Water temperature was controlled by heaters (Lauda E100, with accuracy of ± 0.1 ° C) and a chiller (Aqua medic Titan 1500, with accuracy of ±0.5 °C). Net photosynthesis or respiration rate of corals was calculated in light and dark conditions as the difference in O_2_ concentration between the upstream and downstream chamber multiplied by the flow rate. The measured metabolic fluxes were not affected by gas exchange with the atmosphere since the measurement and sample chambers were in a closed circuit and water was exposed to air only in the source tank.

Autotrophic performance of corals was evaluated by the oxygen anomaly technique, which has the advantage of not being affected by micro-scale variability of photosynthetic activity along surfaces and can be used for estimating net metabolic performance of coral colonies or other macro-photosynthetic organisms (McCloskey, Wethey & Porter, 1978; e.g., Levy et al., 2004). Our semi-closed temperature-regulated aquarium setup illustrated in Fig. 1 is a modified version of the flow-based setups used to measure net photosynthesis of corals in the natural environment and mesocosms (McCloskey, Wethey & Porter, 1978; Jokiel, Jury & Ku’ulei, 2014). The metabolic fluxes were not affected by gas exchange with the atmosphere, since the water was in a closed circuit from the time it was pumped out from the source container until it passed all the measurement and sample chambers and returned to the source tank (Fig. 1).

Prior to experiments, the system was filled with seawater and run for about 2 h until no difference in oxygen concentration between upstream and downstream waters was observed. Throughout the experiments, O_2_ concentrations (mg. l^−1^) in upstream and downstream chambers were simultaneously logged every 5 min using dissolved oxygen loggers (HACH, IntelliCAL™ LDO101 luminescent/optical dissolved oxygen). Oxygen exchange (dO_2_) was calculated by subtracting the downstream O_2_ concentration from the upstream value. The rate of net metabolic performance of corals in our flow-based system was calculated as: Net metabolic rate (mgO_2_. l^−1^. min^−1^) = dO_2_ (mg) × flow rate (l. min^−1^).

Throughout our experiments, the flow rate was nearly constant at about 0.5 l. min^−1^. Net metabolic rate was referred to as net photosynthesis (Pn) when positive as corals were exposed to light, and as dark respiration (R) when negative as corals were kept in darkness. The stability of the response of corals (rate of Pn and R) exposed for ∼2–10 h to constant seasonal temperature levels was tested and confirmed (Supplemental Information). To avoid confounding effects of size-related variability in coral metabolic rates, coral fragments were sampled within a narrow size range (10–15 cm) and inter-subject variability in sample characteristics and performance was accounted for in data analysis (see below).

Light intensity was kept constant at approximately 6500 lux (i.e., about 120 µmol. m^−2^. s^−1^) throughout the experiments (see Supplemental Information). This is close to the average day light intensity measured at the study site in summer: respectively 6,679 ± 6,503 SD and 4,242 ± 3,587 SD at depths of ∼3 m and 6 m (Supplemental Information). Water velocity at the entrance of the chambers (or inside the connecting tubes) was estimated as ∼66 cm. s^−1^ (using 0.5 l. min^−1^ as the flow rate, and ∼4 mm as the diameters of the tubes). This high velocity at the entrance created turbulence and mixed water inside the coral chambers. Water pH was maintained higher than 8 by renewing a constant volume of the source water during the experiments (20 l per 3 h). After the experiments, surviving corals were transplanted back to the sampling site on natural hard substrate or concrete blocks. Living status of transplants was examined subsequently for few months.

### Experiment one: exposure to average and peak seasonal temperatures

In each season, newly collected coral specimens from Hengam reef (*n* = 3–6) were consecutively exposed to 2.5 h periods of light and darkness at the corresponding seasonal mean and extreme temperatures: respectively 23 °C and 20.2 °C in winter, and 32 °C and 34.2 °C in summer. Average rates of net photosynthesis in light (Pn) and respiration in darkness (R) were calculated over the last hour of exposure to each temperature. The net photosynthesis to dark respiration ratio (Pn/R) was calculated as a proxy for the autotrophic capability of corals under each thermal level. Indeed, Pn/R ≥ 1 infers the coral is potentially photoautotrophic with respect to carbon and does not require external supply, while Pn/R <1 indicates that carbon must be acquired from other nutritional sources (McCloskey, Wethey & Porter, 1978). Pn and R were compared among corals exposed to the two thermal levels within each season (seasonal mean vs. seasonal extreme), and Pn/R ratios among corals exposed to the four thermal conditions (summer mean, summer maximum, winter mean, winter minimum) using Generalized Linear Mixed-effect Models (GLMMs). Error terms associated with multiple comparisons on non-independent observations were taken into account by the specification of random effects on subject identity (variability in sample characteristics and performance) in the parameterization of GLMMs, and correction for multiple pairwise Tukey tests (Hothorn, Bretz & Westfall, 2008; Bolker et al., 2009). These statistics were performed in R software (R Development Core Team) complemented by packages ‘nlme’ and ‘multcomp’ at a confidence level of 95% (Hothorn, Bretz & Westfall, 2008; Pinheiro et al., 2008).

### Experiment two: exposure to gradual deviation toward seasonal temperature extremes

Newly collected coral specimens from Hengam reef (*n* = 5) were exposed to gradual deviation in water temperature from the mean annual level of 27.5 °C (temperature of reference, *dT* = 0 °C) toward the seasonal extremes of 38 °C in summer and 17 °C in winter (|*dT*| − 10.5 °C). Temperature was varied at a consistent rate of 0.2 °C per 10 min. This rate is representative of temperature variations as frequently experienced by coral communities on Hengam reefs (Vajed Samiei et al., 2014) and has been widely used in experimental studies to assess thermal tolerance of corals and other animals (Brown & Cossins, 2011). Variation in coral net photosynthesis across temperature ranges was calculated using Generalized Linear Mixed-effect Models (GLMMs). GLMMs are appropriate for analyzing longitudinal data via correction for temporal autocorrelation, and allow for taking into account error terms associated with inter-subject variability via the specification of random effects on subject identity in the model (Bolker et al., 2009). Difference in Pn to *dT* profiles among corals exposed to heating in summer and cooling in winter were established using a modern semi-parametric contrast curve approach combining GLMMs and penalized splines (Durbán et al., 2005). This approach calculates the difference between two curves and allows for the identification of the specific regions of the covariable (here *dT*) where the difference in response variable (here Pn) is significant. The penalized spline component accounts for deviations from the linear model as calculated by GLMMs, while accounting for model complexity and accuracy in an optimal fashion (Ruppert, Wand & Carroll, 2003). Associated with GLMMs and penalized splines, contrast curves thus account for the random effects of comparing longitudinal observations on multiple subjects, and non-linearity in the relationship between the predictor and response variable (Ruppert, Wand & Carroll, 2003; Pinheiro et al., 2008). The underlying mathematics and programming syntax of the contrast curve technique are detailed in Ruppert, Wand & Carroll (2003), Durbán et al. (2005). GLMMs and contrasts were computed in R software (R Development Core Team) supplemented by packages ‘nlme’ (Pinheiro et al., 2008) and ‘BRugs’ (Ruppert, Wand & Carroll, 2003) at a confidence level of 95%. Prior to GLMMs, the relationship between Pn and *dT* was linearized by the square transformation of *dT* to satisfy homogeneity of residuals.

## Results

### Experiment one: exposure to average and peak seasonal temperatures

**Figure 2:**
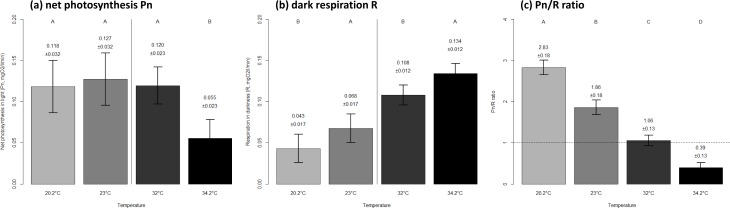
Metabolic performance of corals in constant seasonal temperature levels. Net photosynthesis in light Pn (A), respiration in darkness R (B) and autotrophic capability Pn/R ratio (C) of corals exposed to a range of temperatures as observed on Hengam reefs. The mean ± SE values are indicated on graphs. Letters on top indicate statistically different groups within each season in (A) and (B), and among the four thermal environments in (C). The thermal levels correspond to the winter minimum of 20.2 °C, winter average of 23 °C, summer average of 32 °C, and summer maximum of 34.2 °C.

Coral net photosynthesis was relatively consistent between the seasonal extreme and mean thermal levels of winter (0.12 ± 0.03 versus 0.13 ± 0.03 SE mgO_2_ respectively, *p* = 0.946), while it was considerably lower at the seasonal extreme compared to the mean temperature in summer (0.06 ± 0.02 versus 0.12 ± 0.02 SE mgO_2_ respectively, *p* < 0.0001). Coral respiration was highest at the warmer thermal level within each season (Fig. 2B). The net photosynthesis to dark respiration ratio Pn/R showed a significant decrease with increasing temperature both within and between seasons (Fig. 2C).

All coral specimens survived exposure to the thermal levels tested during the experiments, and no sign of stress was observed one week and six months after transplantation back in the natural environment.

### Experiment two: exposure to gradual deviation toward seasonal temperature extremes

**Figure 3:**
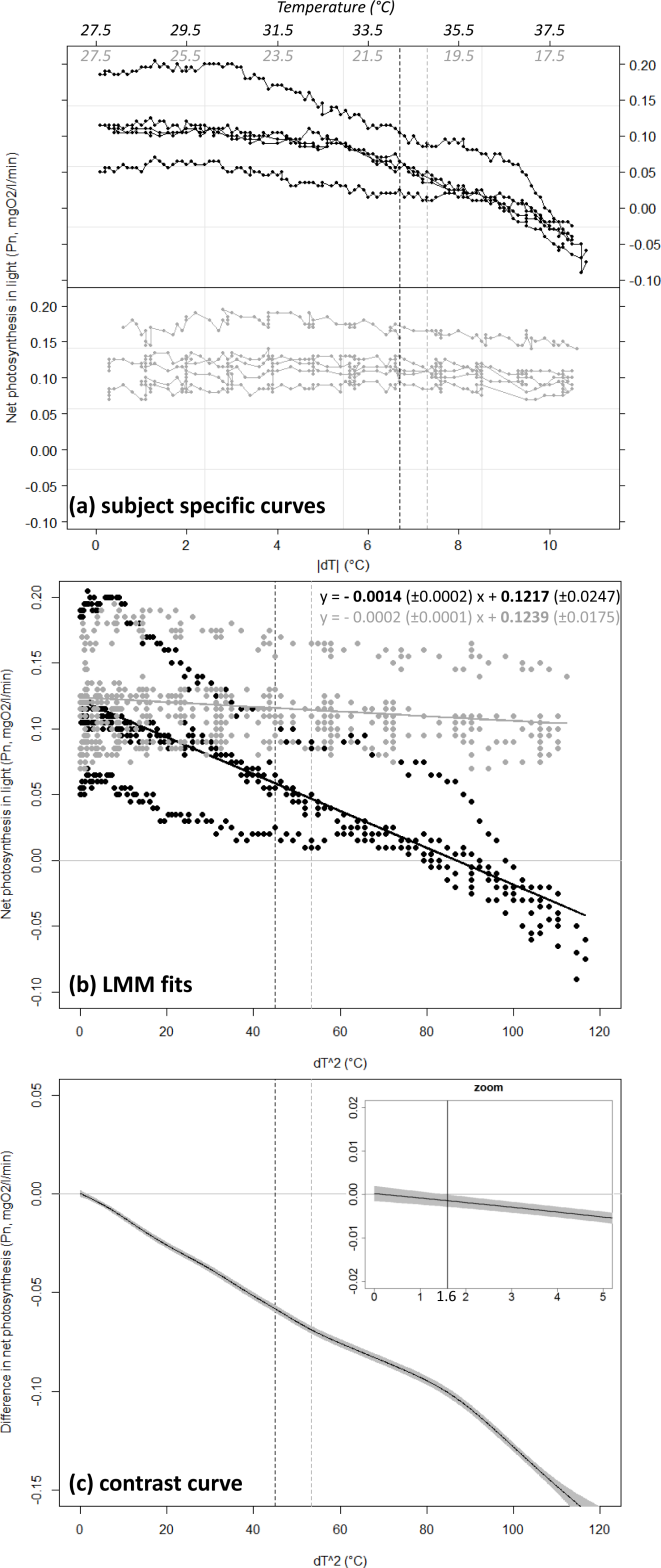
Net photosynthesis of corals exposed to gradual temperature deviations. Coral net photosynthesis Pn as a function of positive (black) versus negative (grey) temperature deviation |*dT*| from the annual mean value of 27.5 °C. Plot (A) shows raw data as recorded for each of the *n* = 5 replicate coral fragments within each treatment. The corresponding temperature ranges are indicated in italic on top of the plot. Plot (B) shows the fit from the Generalized Linear Mixed-effect Model (GLMM) in the linearized dimension (*x* = [*dT*]^2^). The equations of the linear regressions are provided in the form *y* = slope (±SE) *x* + intercept (±SE), and significant equation parameters are printed in bold character. Note the significant negative slope estimated for the summer heating treatment (*p* < 0.001) while the slope is not significantly different from zero in the winter cooling treatment (*p* = 0.171). Plot (C) illustrates results from the semi-parametric contrast curve (based on GLMM and penalized splines) identifying the domain of significant difference between the profiles obtained from the two treatments: the profiles are significantly different when the contrast curve ±CI (black-line ± shading) does not overlap with the *y* = 0 line (here for [*dT*]^2^ > 1. 6 ° C or *dT* = 1.3 °C; see vertical line in zoom insert). Black and grey dashed lines indicate the levels of the peak temperatures observed at the study site in summer (34.2 °C) and winter (20.2 °C), respectively.

Coral net photosynthesis Pn was differently affected by positive versus negative temperature deviations (GLMM, interaction *Treatment* × *dT*^2^, *df* = 1,088, *t*-value = −6.7, *p* < 0.001). Coral Pn remained relatively consistent in winter when water temperature was lowered from the annual mean value of 27.5 °C toward a minimum of 17 °C (estimated Pn of 0.12 ± 0.02 and 0.10 ± 0.01 SE mgO_2_ respectively), but decreased substantially in summer with equivalent positive deviation toward a maxima of 38 °C (estimated Pn of −0.03 ± 0.01 at 38 °C; Fig. 3B). Net photosynthesis was reduced by 50% at the summer maxima level of 34.2 °C (estimated Pn of 0.06 ± 0.01 mgO_2_), and was negative above a temperature of 36.8 °C (*dT* = 9.3 °C). Coral photosynthetic performance differed significantly between summer heating and winter cooling treatments for a temperature deviation |*dT*| > 1.3 °C (*dT*^2^ > 1.6 °C). This domain of significant difference between the two treatments is identified by the contrast curve technique (see Durbán et al., 2005) and illustrated in Fig. 3C as the portion of the covariable ([*dT*]^2^) where the contrast curve and its confidence intervals do not overlap with the no-difference threshold *y* = 0.

Positive temperature-deviations in summer toward annual extremes were associated with decreases in Pn and ultimately with the mortality of corals, while equivalent coolings in winter were not followed by any significant changes in Pn or survival. No sign of stress was observed on surviving coral specimens one week and six months after transplantation back in the natural environment.

## Discussion

Coral net photosynthesis was negatively affected during summer by elevation in water temperature from the annual mean level of 27.5 °C, was reduced by half at the summer maxima of 34.2 °C, and became negative beyond 36.8 °C (i.e., higher O_2_ consumption via respiration than production via photosynthesis). Corals exposed to 38 °C did not survive. In contrast, no significant variation in photosynthetic performance and survival was observed during winter in the face of more than 10 °C decline in temperature.

Previous studies have reported both positive and negative effect of summer temperatures on coral net photosynthesis compared to winter conditions (Ohde & van Woesik, 1999; Bates, Amat & Andersson, 2010; Albright, Langdon & Anthony, 2013; Coles & Jokiel, 1977; Nakamura, Yokohama & Tanaka, 2004; Rodolfo-Metalpa et al., 2010). In theory, coral photosynthesis is expected to show a dome-shaped relationship with water temperature; where the high-value region represents the environmental optimum at which coral performance is maximum (Al-Horani, 2005; Wooldridge, 2014). The position of this optimal thermal environment along the temperature gradient is expected to vary both among species, and among populations of a single species exposed to differing environments, as a result of adaptation and acclimation processes (Coles & Jokiel, 1977). Our results indicate that on Hengam reefs, photosynthetic performance of *Acropora downingi* is limited by the extreme temperatures endured in summer, and thus this environment is above the thermal optimum of the studied population. Previous studies indicated that supra-optimal temperature and high irradiance in summer could be associated with a decrease in photosynthetic efficiency of the symbiont and an increased expenditure of energy in processes involved in protection, chaperoning and repair of the holobiont (Fitt et al., 2000; Downs et al., 2002; Warner et al., 2002; Winters, Loya & Beer, 2006; Suwa, Hirose & Hidaka, 2008). Such extreme environments may also indirectly limit coral performance in other energy-costly processes that are facilitated by photosynthesis such as calcification (Chalker, 1981; Jokiel, Jury & Ku’ulei, 2014; Furla et al., 2000). Indeed, recent studies have shown a temperature-induced decrease in growth of some subtropical corals, including the studied *A. downingi* population of Hengam reef (Vanti, Andersson & Langdon , 2014; J Vajed Samiei, 2014–2015, unpublished data).

Similarly, the balance between net photosynthesis (Pn) and dark respiration (R) displayed by the autotrophic capacity (Pn/R ratio) was lower at summer thermal levels compared to winter ones. Increase in temperature typically elevates the respiration rate of the holobiont as a result of increased metabolic activity, causing a decrease in the autotrophic capacity of the coral (Castillo & Helmuth, 2005). Corals are usually considered autotrophic when the Pn/R ratio is >1, with values <1 indicating higher needs for carbon acquisition through heterotrophy to compensate for losses during respiration (McCloskey, Wethey & Porter, 1978). At extreme conditions, a low autotrophic capacity may restrict the ability of the coral to build and maintain enough energy reserves (e.g., lipids) necessary for sustaining essential homeostatic functions, including sexual reproduction and biophysical stress resistance (Wooldridge, 2014). In our study, corals exposed to the maximum temperature of summer displayed an autotrophic capability ratio below 0.4, suggesting that despite their resistance to bleaching (Vajed Samiei et al., 2014), Hengam reef *A. downingi* are photosynthetically deficient during the warmer summer periods and substantially rely on other ways of energy acquisition.

In this study, we used a constant level of artificial sunlight irradiance that was comparable to the summer daily average irradiance at our study site. In the natural environment, however, daily average irradiance is variable through time and is usually higher in summer compared to winter. In addition, light use efficiency of corals is usually higher in winter comparing with summer (Winters, Loya & Beer, 2006). The seasonality in PAR levels and light use efficiency of corals can induce additional variability in coral net photosynthesis and autotrophic capability between seasons. Therefore, seasonal differences in the reported rates might differ in the natural environment compared to the values observed in our study. In addition, the light spectrum used in our experimental set-up might be somewhat different to that at the collection depth. Interactive effects of temperature deviations and variability in light conditions on autotrophic performance of corals can constitute the focus of future studies using mesocosms or field set-ups. Hengam reef is naturally exposed to tidal temperature variations as large as 5.5 °C in the course of 1 to 3 h (Vajed Samiei et al., 2014). It, therefore, constitutes a suitable site for implementing such studies.

Overall, results from both experiments suggest that *A. downingi* populations around Hengam Island are living closer to their superior thermal threshold, and thus, would be more affected by a warmer climate, or by occurrences of extreme summer events compared to harsher winters. This notably explains why the majority of *Acropora* bleaching events in the highly oscillating thermal environment of the Persian Gulf have occurred during positive temperature anomalies (Riegl, 2002; Sheppard & Loughland, 2002; Riegl, 2003; Burt, Bartholomew & Usseglio, 2008; Riegl et al., 2011), while few bleachings followed negative thermal stresses (Shinn, 1976; Coles & Fadlallah, 1991). Current projections under RCP (Representative Concentration Pathways) 8.5 predict that seawater temperature will increase by 4.26 °C over 2010–2099 in the Persian Gulf (Hoegh-Guldberg et al., 2014). In the current study, coral photosynthetic performance diverged between heated and cooled environments above a thermal deviation of 1.3 °C from the annual mean level, and was rapidly depressed by further temperature increases. *A. downingi* did not survive exposition to a temperature 3.8 °C higher than the peak summer level as experienced in its natural environment. Therefore, in the absence of significant adaptation, forthcoming increase in summer water temperature may strongly impact *A. downingi* populations of the Persian Gulf.

## Supplemental Information

10.7717/peerj.1062/supp-1 Supplemental Information 1 Data of the first experiment

10.7717/peerj.1062/supp-2 Supplemental Information 2 Data of the second experiment

10.7717/peerj.1062/supp-3 Supplemental Information 3 Net photosynthesis of a coral exposed to 32C for more than 10 h

10.7717/peerj.1062/supp-4 Supplemental Information 4 Light intensity (lux) data collected in Hengam coral patches at depths of about 3 and 6 m during the warm season

10.7717/peerj.1062/supp-5 Supplemental Information 5 Exemplary data set indicating the light intensity during the experiments
